# Secured Perimeter with Electromagnetic Detection and Tracking with Drone Embedded and Static Cameras

**DOI:** 10.3390/s21217379

**Published:** 2021-11-06

**Authors:** Pedro Teixidó, Juan Antonio Gómez-Galán, Rafael Caballero, Francisco J. Pérez-Grau, José M. Hinojo-Montero, Fernando Muñoz-Chavero, Juan Aponte

**Affiliations:** 1 OnTech Security LLC, C/Hispano Aviación, 7-9, 41300 Seville, Spain; pedro.teixido@ontech.es (P.T.); juan.aponte@ontech.es (J.A.); 2 Departamento de Ingeniería Electrónica, Sistemas Informáticos y Automática, Universidad de Huelva, 21007 Huelva, Spain; 3 Advanced Center for Aerospace Technologies, C/Wilbur y Orville Wright 17-19-21, 41300 Seville, Spain; rcaballero@catec.aero (R.C.); fjperez@catec.aero (F.J.P.-G.); 4 Departamento de Ingeniería Electrónica, ETS Ingenieros, Universidad de Sevilla, 41092 Seville, Spain; jhinojo@us.es (J.M.H.-M.); fmunoz@us.es (F.M.-C.)

**Keywords:** perimeter, detector, preventive, intrusion, alarm, cameras, drone, CEMF

## Abstract

Perimeter detection systems detect intruders penetrating protected areas, but modern solutions require the combination of smart detectors, information networks and controlling software to reduce false alarms and extend detection range. The current solutions available to secure a perimeter (infrared and motion sensors, fiber optics, cameras, radar, among others) have several problems, such as sensitivity to weather conditions or the high failure alarm rate that forces the need for human supervision. The system exposed in this paper overcomes these problems by combining a perimeter security system based on CEMF (control of electromagnetic fields) sensing technology, a set of video cameras that remain powered off except when an event has been detected. An autonomous drone is also informed where the event has been initially detected. Then, it flies through computer vision to follow the intruder for as long as they remain within the perimeter. This paper covers a detailed view of how all three components cooperate in harmony to protect a perimeter effectively, without having to worry about false alarms, blinding due to weather conditions, clearance areas, or privacy issues. The system also provides extra information of where the intruder is or has been, at all times, no matter whether they have become mixed up with more people or not during the attack.

## 1. Introduction

In recent years, intrusion detection sensing systems have expanded significantly to meet the growing demand for improved security. Increasing investments for infrastructure development have widened the scope for the growth of the perimeter security market. Between 2014 and 2025, around 78 trillion USD is projected to be spent on infrastructure development worldwide [1].

Reducing the vulnerabilities of critical infrastructure and increasing its resilience is one of the primary objectives of the European Union (EU). The European Programme for Critical Infrastructure Protection (EPCIP) sets the overall framework for activities to improve the protection of critical infrastructures in Europe—across all EU States and in all relevant sectors of economic activity [2]. With increasing security threats in Europe and worldwide, organizations managing critical infrastructures such as hydroelectric, solar, nuclear, or thermal power plants and oil refineries or wastewater treatment plants have a growing need for enhanced perimeter protection systems with reduced false alarm rates and preventive detection capabilities.

Modern integrated surveillance and security management systems require the implementation of smart detectors, information networks and controlling software. While only “real” alarms, representing threats, should be detected by the sensors in an ideal case, the reality is that the devices generate many unnecessary warnings. These events can be classified as false alarms or as nuisance alarms. False alarms are produced by events that should not trigger an alarm. On the other hand, nuisance alarms are generated by a legitimate cause, but without it representing a real threat. In electronic security systems for critical facilities and infrastructure protection, the sensors most frequently used by the intrusion detection subsystems are the passive and active infrared, accelerometer, microwave, ultrasonic, optical fiber (FBG) sensors and perimeter protection based on buried sensors [3,4,5,6,7,8]. Infrared and motion sensors suffer severe range reduction in rain and fog. They are limited to direct line of sight detection and can be triggered by any kind of moving object. Fiber-optic and microphonic cable detection systems suffer from elevated fault alarm rates. Ground radar systems, microwave barriers and buried systems require a large clearance area free of vegetation and obstacles to operate properly. Camera-based systems usually require human supervision, and they are often in conflict with the GDPR. Electrostatic field disturbance sensors have a nearly zero fault alarm rate but cannot track the intruder once inside the property. Therefore, the efficacy of most of them is often limited by high false alarm rates (>40% false alarms, on average) and the detection range.

Regarding the electrostatic field sensors, it is a known approach of capacitive sensing based on the disturbance in the electric field created between a driving electrode and a sensing electrode [9,10]. Capacitive sensing has gained increasing importance in the last decades and is successfully employed in various applications in industrial and automotive technologies because it involves low-cost, design simplicity, high sensitivity, no direct contact with the measured object, and frequency stability [11]. Several works have been reported on this detection principle [12,13,14,15,16]. Seat occupancy detection for air-bag control in vehicles based on electrostatic field sensing has been presented in [12], providing information about the presence, type and position of an occupant using eleven transmitting electrodes and a common receiving electrode. In [14], a capacitive proximity sensor based on the transmit mode for the application of human−computer interaction is presented. A combination of inductive and capacitive sensing modes can help to distinguish different objects and obtain distance information [15], since inductive sensors are a recognized technology for sensing metallic objects, yet their sensitivity to materials that possess low conductivities or are nonmagnetic, such as the human body, are much lower [16].

The integration of multiple intrusion-detection technologies into a given sensing solution provides it with better robustness and reliability when compared with approaches based on a single technology. However, attempting to reduce false alarms can be detrimental to detection probability. On the other hand, camera systems tend to be the preferred solution for the security systems of critical facilities. They present high costs in both operation and maintenance, while having a low probability of preventing threats. This low probability is related to the system dependency of threat identification on the operator’s recognition of the camera images presented on various screens and the cabling needs of the devices, which can generate restrictions on the areas that can be monitored. Some solutions propose a multiple-sensor platform with video analysis to detect and classify mobile objects [17,18,19,20]. These technologies are not power efficient in operation because sensors and cameras must always be operational to be prepared for a possible intrusion (resulting in a 60–80% energy inefficiency).

The proposed approach aims to achieve a scalable and cost-effective security solution to be deployed on large perimeters of critical infrastructure with reduced operational costs since it can reduce the amount of video monitoring data to be processed and decrease the workforce costs to the automatic intruder tracking. The CEMF technology comes into play with a more extended detection range (20 m/sensor) and reduced false alarms (<10%) while being able to work on all terrains and independent of weather conditions. This technology can differentiate between different types of events, objects, animals, or persons. This ability enables a preventive feature that allows it to generate an alarm before an intrusion occurs. Finally, as cameras remain inoperative except when an event is detected in its field of view, the system decreases power consumption by reducing the use of video surveillance by around 95.8% in comparison to other systems (none of them can deactivate the video surveillance), as well as the amount of data that has to be saved in data centers.

Across this study, a detailed view on how the disruptive CEMF technology works is obtained, and how the usage of an ensemble that joins traditional algorithms, a random forest and a neuronal network, can provide a great level of discrimination between events. Such achievement allows for a sophisticated video tracking system to be triggered on any dangerous alarm. This complementary video tracking system combines fixed cameras and a drone embarked camera to provide a continuous stream of video showing where the intruder is at all times, no matter whether they have moved away from the fixed cameras. This paper also describes the different techniques used to analyze video, and how the algorithms used for fixed cameras differ from the ones used for the drone camera. This combination of the novel CEMF discriminative perimeter technology, plus a highly innovative camera system presents an unprecedented security system.

## 2. System Overview

The proposed system integrates three main parts. A high-performance perimeter sensing network based on CEMF and capable of detecting and differentiating preventive and intrusive events. A fixed camera network, installed along the perimeter that remains off at all times except when the sensing network detects an event, a moment in which only the camera/s covering that location turn on to send the video data to the server. An unmanned aerial vehicle (UAV) reacts to the detection of an event by flying to the intruder’s location and follows the intruder during the attack, providing video data even when the intruder has walked away from the perimeter. The rest of the necessary parts to complete the system: a local server in charge of processing the video streams to perform the intruder tracking and request cameras to switch on/off to maintain the maximum number of cameras powered off while ensuring video coverage of the intruder at all times; a cloud server to store a large dataset from which to supply information to a machine learning algorithm; a monitoring station to communicate events to the operator and provide video data of the active cameras.

**Figure 1:**
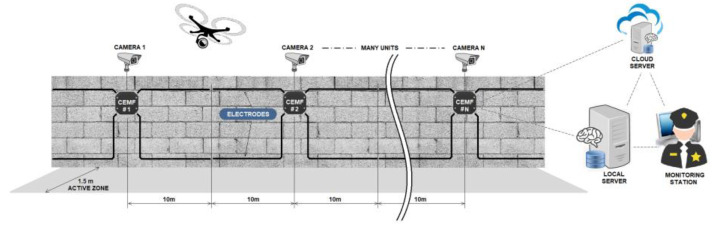
System overview.

In Figure 1, the six main elements of the proposed solution are represented. Although only three CEMF units are drawn, there can be as many units as required to cover the entire perimeter in a real installation. The electrodes are arranged at two different heights to cover the entire perimeter. The top-level expands its electromagnetic field over the fence, while the low level covers the lower part. The active detection zone extends 1.5 m away from both electrode levels. The units are separated, 20 m from each other (10 m per electrode), and a camera can be optionally installed with each perimeter unit. The cameras and the UAV are activated with the location of the CEMF sensor triggered by the intruder. Both systems provide video streaming of the intruder to the local server and the monitoring station that can be located at any place where an intranet connection is available. In contrast, a connection to the internet is required to connect to the remote cloud server.

## 3. CEMF Secured Perimeter

The basics of the CEMF sensing technology are the simultaneous generation, control, and monitoring of an electromagnetic field. This characteristic has been developed in a highly innovative technology capable of sensing tiny changes within its electromagnetic field lines. This technology can be easily adapted to work on industrial security, explicitly targeting critical infrastructures, enabling the surveillance of large perimeters.

The principle of operation relies on the fact that the human body exhibits its own electrical characteristics like any other existing object. Electromagnetic charge migrates from one object to another when they interact, causing changes in the field. Thus, the CEMF technology continuously measures these differences. It can distinguish and classify different types of events and discriminate between the presence of humans, animals or objects by analyzing the profile generated by the event. This property enables the system to detect intruders while ignoring animals or other inoffensive events. In the same way, the system can distinguish whether a human is standing next to the fence or is climbing it, creating a truly preventive and intrusive perimeter sensor.

The proposed solution has specific characteristics that make this device a unique one:

(a) *Invisibility*: The electromagnetic field generated crosses objects and walls (the frequency of operation is in the range of kHz), allowing the sensor to be hidden behind or even inside a wall, which is not possible with the conventional IR or microwave sensors.
(b) *Long Range*: The sensor can detect an object up to 10−20 m along the length of a wall and 1.5 m beyond the breadth of it.
(c) *Discriminative*: The technology can detect the perturbation corresponding to a person’s disruption in the electromagnetic field, different from an object or even an animal perturbation. As a result, the sensor can distinguish between a person and any other type of intruder, thus reducing false alarms from 40% (other technologies) to 10%.
(d) *Preventive detection*: Threats are detected even before any harmful actions occur, responding with different alarm levels. The grade of the threat can be measured by distinguishing between loitering, touching, or climbing the fence without the need of cameras.
(e) *Tracking and identifying the intruders:* As an intruder moves along the wall, the units track its route along the perimeter.
(f) *Personal data protection law-friendly*: The cameras can remain switched off most of the time and switch back on in the event of an alarm.

### 3.1. Architecture

**Figure 2:**
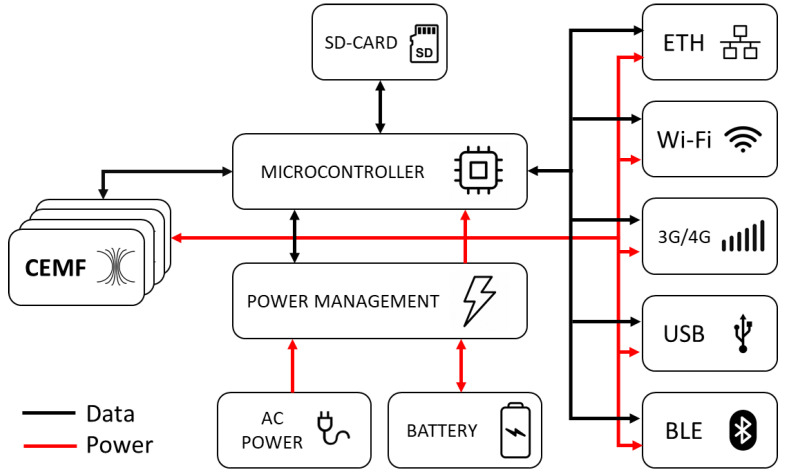
CEMF sensor architecture.

The hardware was developed according to its size efficiency, low cost, and low power usage requirements. Power consumption is critical because the intrusion system regulation UNE 50,131 requires devices to have a battery life of at least 30 h. Lowering the power consumption reduces the battery capacity, which results in a smaller device size and a lower cost. Figure 2 shows the hardware overview diagram of the system.

The system board has four independent CEMF channels, and it performs field generation, data acquisition, signal conditioning, algorithm, communications (both wired and wireless), data storage and power management. A 32-bit PIC32MZ2048 microprocessor from Microchip governs the system, capable of running up to 200 MHz and with 120 I/O pins (48 analog), UART, SPI, I2C and USB as well as an embedded real-time clock (RTC). The usage of the Microchip PIC32 was decided in order to reuse the know-how, tooling and a set of stable libraries already developed and validated. Then, within the PIC32 family, a large memory (flash and ram) and DSP core were required, as neuronal networks had to be executed. A large number of GPIOs were also required to control all the devices and acquire four CEMF channels as well as an Ethernet MAC to provide connectivity. At the time of the architectural design, when all the microcontroller’s requirements were applied, the PIC32MZ2048 was the clear choice taking into account the lead time, compatible parts as well as pricing.

The communications have been designed in a redundant way to avoid losing connectivity, and thus, the mainboard incorporates the following interfaces: Ethernet, Wi-Fi, mobile data (3G/4G), USB and Bluetooth. The first three provide connectivity to the local/cloud server. Ethernet is the preferred method, followed by Wi-Fi and 3G/4G. The other two interfaces are designed to provide in situ maintenance, so that a technician can connect and configure/debug the unit during installation or failure analysis. The board also has an SD card that can be used for data recording and firmware upgrades on isolated units. A group of four relays with latch functions is used to communicate with legacy security systems. The user can configure their behavior in terms of NO/NC output and concept mapped to each relay.

The system is supplied with AC voltage converted into 5 V DC by a flyback power supply. Several DC/DC converters have been used to achieve the 3.3 V required to supply most digital circuits, 18V for the operation of the CEMF sensors, 1.2 V for the Ethernet switch and 3.8 V for the 3G communications. The power management module also incorporates a battery charger able to handle a large LiPo battery of 10 Ah and 3.7 V calculated to provide enough energy to continue operating for up to 30 h. This battery ensures the unit never loses connection and sends alerts even if the power grid is disconnected in a power outage.

Finally, the device includes several tamper sensors to detect manipulation attempts. An accelerometer to detect position changes, a standard switch to detect the enclosure aperture, and a hall sensor to detect detachment from its mounting plate with an integrated magnet.

**Figure 3:**
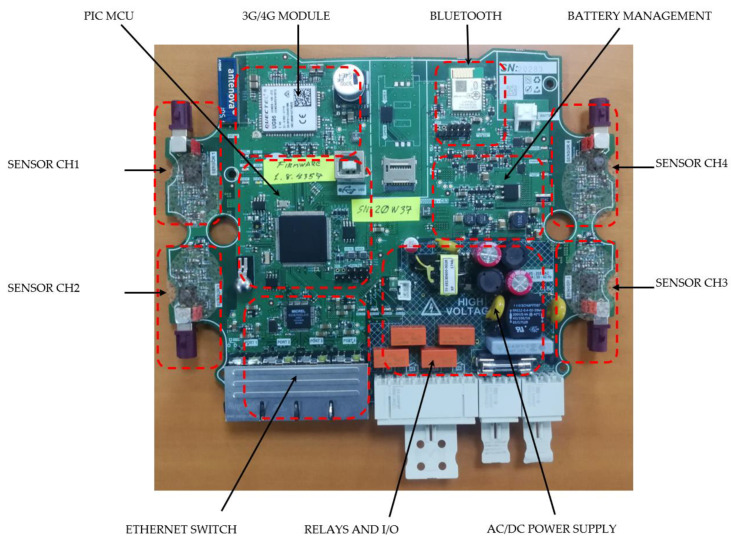
CEMF sensor board.

**Figure 4:**
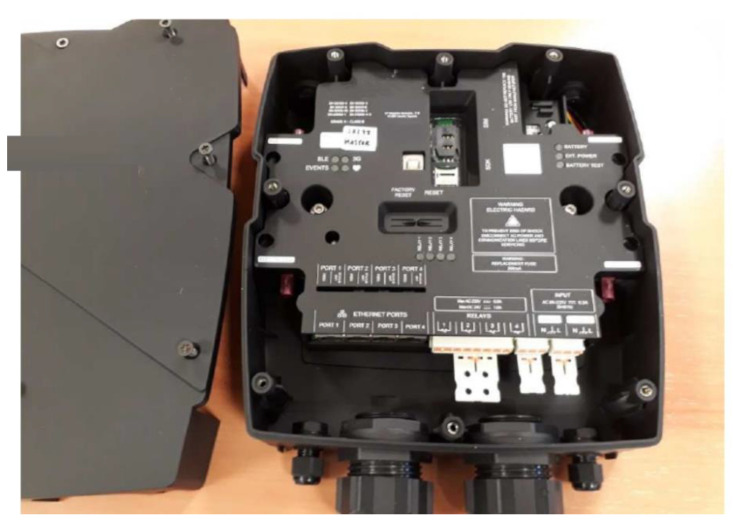
CEMF sensor inside its enclosure.

Figure 3 shows the CEMF sensor board where the main blocks have been highlighted to provide a clear understanding of block size and complexity. Figure 4 shows the same board installed inside its enclosure (enclosure open, see a part of the lid on the left of the picture).

### 3.2. Algorithms

**Figure 5:**
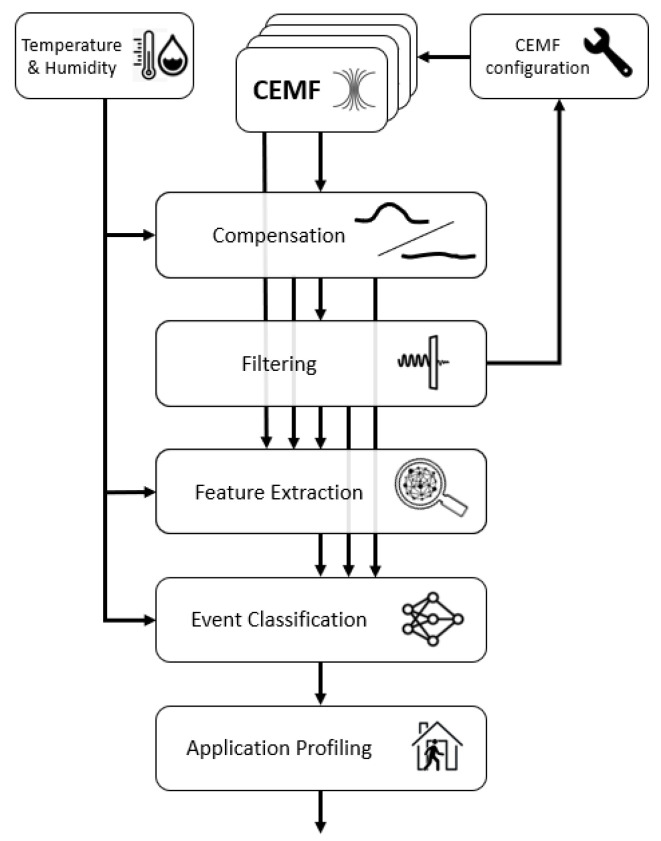
CEMF algorithm diagram.

The performance of the CEMF technology relies on both hardware and algorithms. The algorithms are responsible for setting the resolution, sample rate, power consumption, raw signal noise, compensation, filtering, feature extraction, event classification, and application profiling to fulfil the application policies such as the enable, disable, arm or guard times. Figure 5 shows how the data flows across the different blocks of the algorithm to generate the algorithm output; this section covers, one by one, each of the blocks here stated.

The most critical CEMF configuration settings are resolution and sample rate. These define the sensitivity, noise and power consumption of the system. Other parameters, such as start and stop times, can be adjusted to fine tune the result.

A thorough characterization has been performed to ensure the correct value of these parameters to fit the application requirements. This characterization considers a large number of different approximations of an object for calibration to the electrode. With the data collected throughout the experiments, a statistical study is conducted to understand the performance of the raw CEMF technology. These steps have been repeated several times until the best performance has been found.

**Figure 6:**
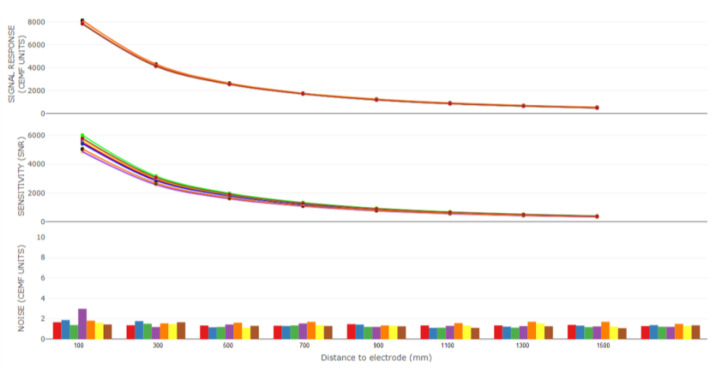
CEMF characterization.

Figure 6 shows the CEMF signal along with one of these iterations. The top graph represents raw sensitivity in CEMF units vs. distance. The second graph is the SNR sensitivity vs. distance, and the last one represents the noise vs. distance in the CEMF units.

As shown on the diagram, the CEMF data starts its journey through the *Compensation* block. It uses temperature and humidity information plus some of the CEMF data to compensate for the CEMF readings. This process guarantees that data from any sensor look approximately the same regardless of whether it is installed in a rainy, cold or dry location. The same applies to fast and irregular changes due to direct sun and shadow transitions or rain, or any environmental change that could occur to the sensor.

**Figure 7:**
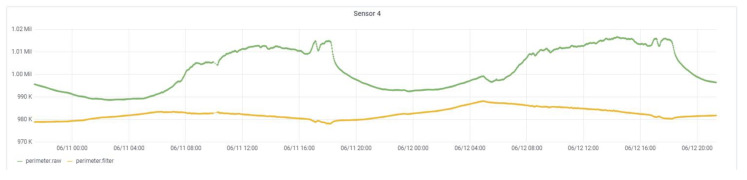
CEMF compensated signal.

An exponential weighted moving average (EWMA) low-pass filter was chosen to eliminate undesired noise and focus on the recent past as shown in Figure 7. The magic of this filter is assigning a weight to each acquired sample, the weight decreases in an exponential way for previous samples. This feature not only gives greater importance to recent events, but also removes the necessity of storing infinite values, as at some point their weight is so small that they can be just ignored, as a result, the EWMA can be calculated with a fixed buffer size.

The EWMA can be obtained from the SMA formula (see Equation (1)). The SMA can be expressed as shown in (1) where $\mu_{i}$ symbolizes the moving average at point $x_{i}$. The variance of the SMA is shown in (2).

(1) $$\mu_{i}^{SMA}=\frac{1}{n}\sum_{i=0}^{n}x_{i}$$

(2) $$\sigma^{2}=\frac{1}{n}\sum_{i=1}^{n}{\left(x_{i}-\mu \right)}^{2}$$

To obtain a weighted moving average (WMA) from the SMA, weights (w) can be given to previous points used to obtain the mean. Equation (3) shows the formulation of WMA, where $w_{i}$ express the weight at point $x_{i}$. The variance of the WMA is shown in (4).

(3) $$\mu_{i}^{WMA}=\frac{w_{i}x_{i}+w_{i-1}x_{i-1}+w_{i-2}x_{i-2}+\ldots +w_{i-n}x_{i-n}}{\sum_{i=0}^{n}w_{i}}=\frac{\sum_{i=0}^{n}w_{i}x_{i}}{\sum_{i=0}^{n}w_{i}}$$

(4) $$\sigma^{2}=\frac{\sum_{i=1}^{n}w_{i}{\left(x_{i}-\mu \right)}^{2}}{\sum_{i=0}^{n}w_{i}}$$

If the weights are defined as (5), we get the EWMA where the weights decrease in an exponential way for previous values. Thus, the EWMA is presented in (6).

(5) $$w_{n}={\left(1-\alpha \right)}^{n}$$

(6) $$\mu_{i}^{EWMA}=\frac{x_{i}+x_{i-1}\left(1-\alpha \right)+x_{i-2}{\left(1-\alpha \right)}^{2}+\ldots +x_{i-n}{\left(1-\alpha \right)}^{n}}{\sum_{i=0}^{n}{\left(1-\alpha \right)}^{i}}=\frac{\sum_{i=0}^{n}x_{i}{\left(1-\alpha \right)}^{i}}{\sum_{i=0}^{n}{\left(1-\alpha \right)}^{i}}$$

In addition, the variance for the EWMA is shown in (7).

(7) $$\sigma^{2}=\frac{\sum_{i=1}^{n}{\left(1-\alpha \right)}^{i}{\left(x_{i}-\mu \right)}^{2}}{\sum_{i=0}^{n}{\left(1-\alpha \right)}^{2}}$$

As can be seen, the weights are defined by *α*, which defines the depth of the filter. The value of alpha will result in an hyperparameter for the implementation, that needs to be adjusted. As a rule of thumb, the value of alpha that gives a high weight to n number of previous samples can be obtained using (8).

(8) $$\alpha =\frac{2}{n+1}$$

**Figure 8:**
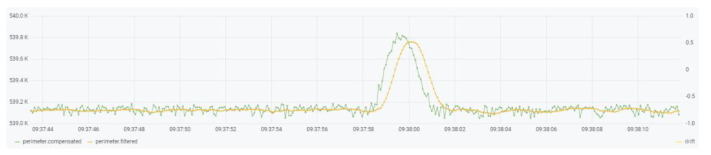
EWMA filter performance.

Figure 8 demonstrates the performance of the filter implementation with actual data captured and filtered on the device. The green represents raw data and is the input of the filter, while the yellow represents the filtered data delivered at the output of the filter. Observe how the high frequency changes produced on the input signal are not present on the output.

The following stage extracts features from all CEMF channels and their relationships. These features help the classifier to separate events that may appear similar by looking only at the CEMF signals directly:

- Correlation for left−left, right−right, top−top, bottom−bottom and all channels;
- Maximum and minimum for all channels;
- Skewness for all channels;
- Standard deviation envelope for all channels;
- Variance for all channels;
- Derivative signal for all channels (differentiation).

**Figure 9:**
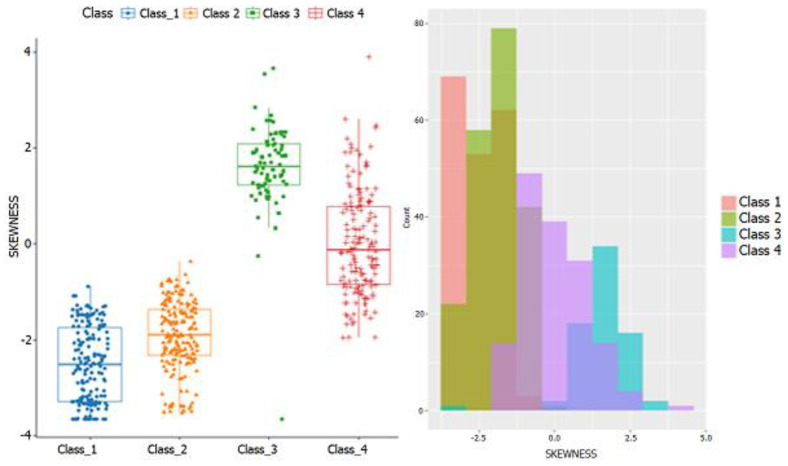
Example of feature extraction (skewness).

Figure 9 shows an example of the skewness analysis. Observe how this analysis helps to separate classes 1 and 2 from classes 3 and 4. Other features help separate different classes, providing different points of view to help the classifier increase its performance.

All these features are continuously extracted and feed into the classifier for more accurate detection. The classifier has been implemented as an ensemble of three different techniques. All of them contribute to increase the number of true positives and reduce the number of false negatives. The techniques used are random forest, artificial neural network (ANN) and traditionally based on thresholds.

**Figure 10:**
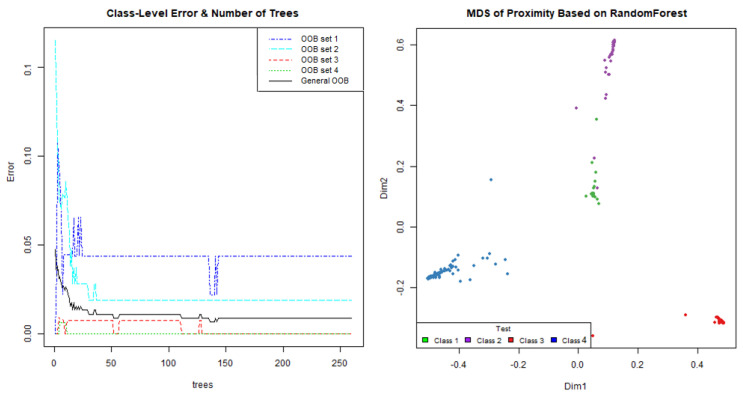
Random forest OOB analysis and final class separation.

A multiclass random forest has been implemented to distinguish between four classes: idle, prowling, intrusion and cars. The number of trees in the model has been optimized to reduce the memory footprint as much as possible. For this, the OOB (out of bag) classification error of the model with respect to the number of trees has been used. As shown in Figure 10, the OOB remains constant from 100 trees onwards. In the right graph, a representation of the final separation made by the random forest is shown.

**Figure 11:**
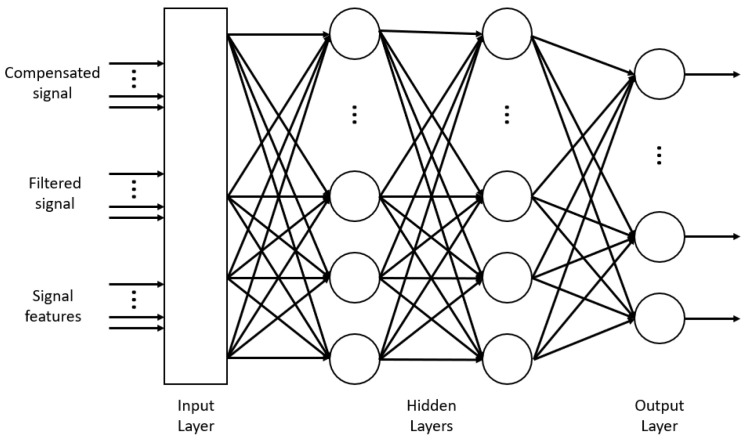
ANN diagram of the CEMF algorithm.

The second classifier is an ANN network. The input and output layer are fixed to input parameters and output values, respectively. As output can take four values only (idle, prowling, intrusion and car), four neurons were used. The input layer takes the compensated and filtered signal plus the six features mentioned above for each one of the four channels. The signals are passed through a max-pooling process in order to down-sample the vectors to four values each. Therefore, the input layer has 56 inputs (plus bias). As input and output are fixed, the number of hidden neurons is the main hyperparameter to optimize. It was important to consider that the complexity of the algorithm grows exponentially with the number of neurons and layers, but its accuracy also increases with it. In this case, the best obtained tradeoff was considering only one dense hidden layer with seven neurons. This number is small enough to be used in real time in the PIC32, which was the hardest restriction for this block. The outputs are softmax nodes, as many works have demonstrated that it offers the best performance for categorical classification, where only one output neuron has to be active for each input pattern. The rest of the neurons of the network use tanh function. Figure 11 represents the diagram of the described ANN.

The last part of the ensemble is the traditional algorithm that has been coded using a set of thresholds and hysteresis distributed on the different CEMF channels and the extracted features. These thresholds, hysteresis and other parameters are dynamically enabled, disabled, or adjusted through a set of relationships between them and the input signals. The result is an adaptive algorithm capable of delivering good performance with low computing power.

The combination of the three techniques provides a very accurate classification of events. In Table 1, it is possible to see how the algorithm has performed in the test dataset. The dataset was built using some of the installed units considered training units. These units work together with continuous video recording of the perimeter they protect. Both CEMF and video data is continuously pushed to a private database. In the server side, a very simple algorithm analyzes the CEMF stream looking for any kind of activity in the signal. The server labels the events as unclassified. Later on, a custom application presents the CEMF pattern and the video in a clean UI interface used to manually review and classify all unclassified events. Once the event is classified, a metadata record is stored in a MongoDB where the training and validation dataset are hold.

Accuracy on intrusion and car detection is 100%, while the performance between idle and prowling is a little bit lower. In particular, one idle test, corresponding to a rainy and windy capture, has been misclassified as prowling. The other scenario means that 7.3% of the prowling events have been classified as idle. This has been balanced by design, as the priority was to reduce the false alarm rate to the minimum.

## 4. Camera Network System

### 4.1. Architecture

The goal of the camera network system is to monitor a configured area with the cooperation of the CEMF sensor network. It makes use of computer vision algorithms to detect intruders and track them. All the components are connected through a local network with static IP addresses using Ethernet cables. Different communication protocols are used on this network depending on what should be transmitted. The camera network system is composed by:

- Cameras: IP cameras were selected since they are a very extended surveillance solution (almost a standard) and also for the characteristics that they may offer. Specifically, the chosen camera is the HIKVISION Network Cam Model DS-2CD2055FWD. Among all its features, it is important to highlight its capacity of changing resolution and other characteristics such as the wide range of supported protocols (HTTP, RTSP…). It is waterproof, making it compatible with an outdoor system. It has a graphical web interface via HTTP where all the parameters can be set. For this work, a resolution of 640p has been set. The frame rate was set to 12 fps to reduce the network load and the required processing. Regarding the installation position, it is advisable to place them at a minimum height of 3 m and point them slightly downwards to help the computer vision algorithm perform its task.
- Data processing unit: The data processing unit is where all the information is processed. Due to the high computing needs required by computer vision algorithms, an Intel NUC was used. Of course, just one Intel NUC does not have enough computing power to process the video stream of many cameras simultaneously. Because of this, the system is scalable with extra processing units to distribute the load. Currently, each Intel NUC can simultaneously handle the load of five cameras.
- External monitoring unit: Basically, it is a PC connected to the network to receive the output video broadcast by the data processing units. This device must be configured in the same IP domain range and have some generic software (such as a web browser…) that can reproduce HTTP streams.

**Figure 12:**
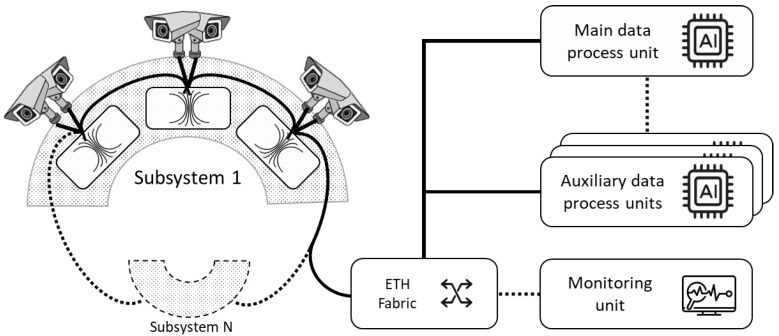
Video-tracking system scheme.

The general system scheme explained above is shown in Figure 12. As can be seen, every device is connected with each other through the Ethernet fabric. The dashed lines between IP cameras, processing units and subsystems represent the possibility of introducing new devices of the same type. The dashed line connecting the Ethernet fabric with the monitoring unit indicates that the system can perfectly operate without it, making it an optional complement that does not impact the system if, for any reason, it fails.

### 4.2. Algorithms

As mentioned in 4.1, the processing units are in charge of managing the entire video stream and alarms generated by the CEMF perimeter, tracking the intruder, and extracting the video stream of the camera with the best view of the intruder. Each processing unit uses Ubuntu version 16.04 LTS as the operating system. The development is carried out using C++ and Python programming languages. In addition, specific external libraries and frameworks were used to facilitate the development and tuning of the system. Among them, the most important are OpenCV and ROS (Robot Operating System).

**Figure 13:**
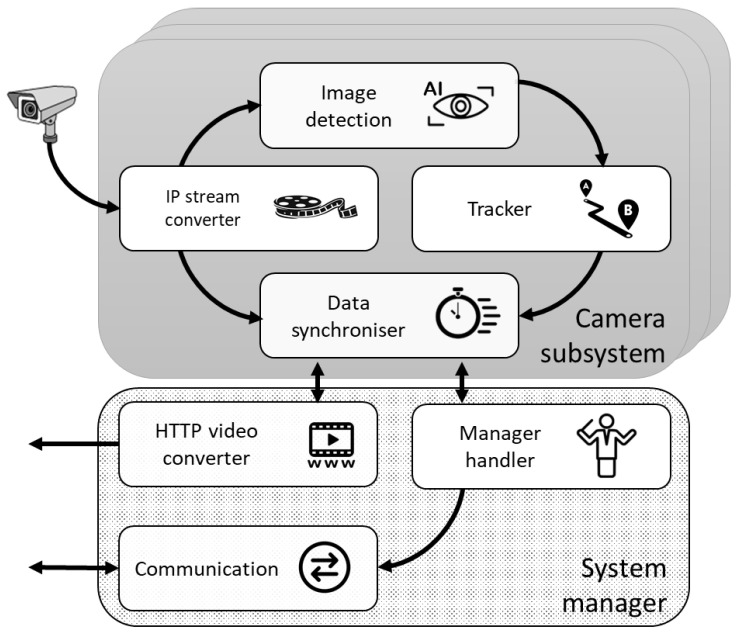
Camera network system—overview.

The software system was developed with a multiprocessing concurrent pattern in which each process has a specific function. This pattern helps optimize the processor load and improves overall performance. Each process is known as a node and uses the ROS framework to deploy and communicate with others. Figure 13 shows a diagram with the primary nodes of the system.

Only one *System manager* runs in the main data processing unit, while the *Camera subsystem* is replicated once per camera and can be instantiated in any data processing unit. The function of each node is as follows:

#### 4.2.1. Camera Subsystem

**Figure 14:**
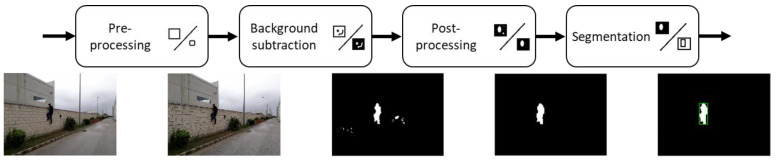
Image detection pipeline.

a. IP Stream Converter. This node is responsible for establishing a connection with its associated IP camera. The camera sends the frames to this node through the RTSP protocol, converting them to a format suitable for processing by the rest of the nodes. If the connection between the camera and the node is interrupted or cannot be established, the process keeps trying to establish the connection.
b. Image Detection. It receives frames from the *IP Stream Converter* and processes them to extract the detections. This process with the heaviest load is performed by a pipeline with the algorithm stages shown in Figure 14.

**Figure 15:**
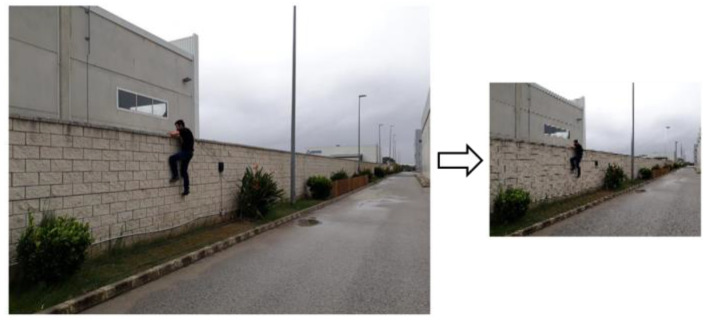
Image detection—preprocessing.

*Preprocessing*. The first step is the preprocessing stage. It uses an edge-preserving smoothing technique to reduce resolution, accelerate the image process, and clean up the image to avoid pixel noise impacting the next pipeline stage (Figure 15).

**Figure 16:**
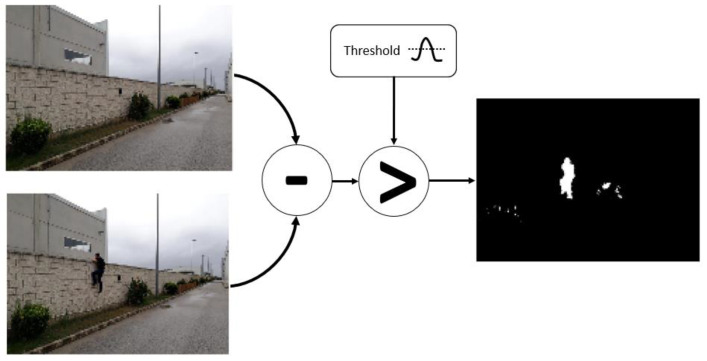
Image detection—background subtraction.

*Background subtraction*. After preprocessing has been done, a foreground object detection technique is used to remove static parts of the image. The technique used is MOG (mixture of Gaussians) [21,22]. This algorithm aims to apply background subtraction to detect shadows and just background and foreground pixels (Figure 16). It creates a Gaussian model for each one of them. As it considers small object movements as part of the model, a longer training time is required. This tolerance to small changes makes it more reliable in a range of more realistic situations, such as changes in light or movements of branches of a tree.

**Figure 17:**
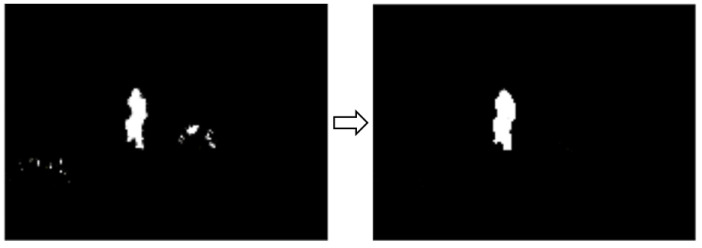
Image detection—postprocessing.

*Postprocessing.* The result of the background removal step is far from ideal. Different morphological operations can be performed on the image, such as dilation or erosion. The dilation operation looks for white areas in the image and expands their edges to group regions of interest. On the other hand, the erosion performs the inverse operation, reducing the small regions that do not provide relevant information (Figure 17).

**Figure 18:**
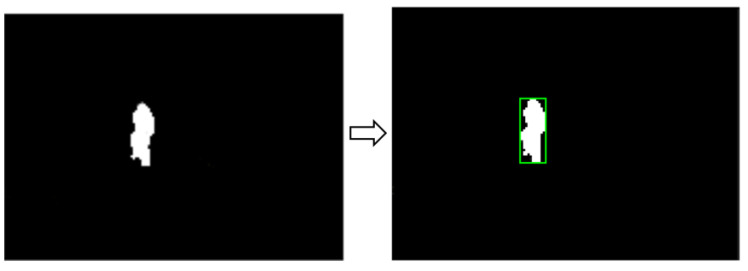
Image detection—segmentation.

*Segmentation.* Contour detection is the technique [23] that has been applied to the output of the postprocessing to detect and locate where activity is taking place. Over the image, a previous black and white image are used, and a process is applied to find the contours and frame them in identifying rectangles (Figure 18).

c. Tracker. This process is in charge of processing the events generated by the *image detection* node and establish a relationship between them. This node has to make decisions based on different characteristics such as the lifetime, size and position of the detections or their speed. When a detection vanishes (e.g., such as the intruder walking behind a column), a Kalman filter is used to estimate the current position of the intruder and maintain a tracking until the *Image Detection* can locate the intruder again. Another important point is the activation of nearby cameras when an intruder leaves the field of view of an active camera towards that of an inactive camera.
d. Data Synchronizer. This node merges the images and detections, creating an output video stream with the processed information of the detections. This video is sent to the HTTP Video Converter.

#### 4.2.2. System Manager

a. Comms. Communicates with the CEMF sensors. It creates a virtual map that is kept updated with each message. This information is then served to the *manager handler*.
b. Manager Handler. This is the first node to boot. It reads the configuration file that includes IP addresses and ports of the cameras and processing units and the information about how everything is related. All that data starts the *communications* node and all the required *data processing units* and boots the *camera subsystems*.
c. *HTTP* Video Converter. This node converts the output video and executes an HTTP server where connections can be made, and the video can be viewed in a standard web browser.

## 5. Autonomous Drone System

The autonomous drone system aims to provide a video stream of the intruder when the ground security cameras are out of reach.

**Figure 19:**
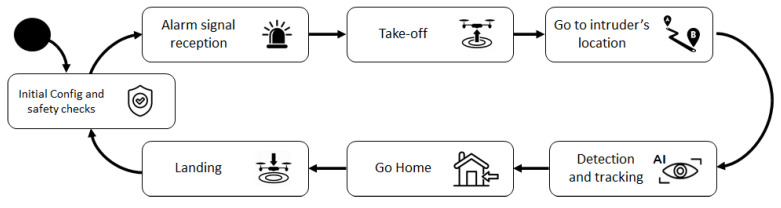
High-level mission state machine.

Figure 19 shows the diagram of the high-level state machine in charge of executing the missions. The initial state assumes the system remains in the idle state just after initial configuration and safety checks. When any CEMF perimeter sensor detects an alarm, the drone automatically takes off. The ground system communicates the intruder geolocation to the drone, which initially knows the location of the CEMF sensor that detected the event. While the drone is flying to this location, the ground camera system keeps track of the intruder and updates its location (see Section 4 for details). Once the drone reaches the intruder location, it keeps track of it. Once the mission is complete, the drone goes back to the take-off position and lands.

The ground system is always updated with the geolocation of the drone itself and the intruder’s location when available.

### 5.1. Architecture

One critical point to guarantee the success of this block was the right selection of the drone. Therefore, one of these leading players had to be chosen:

- DJI;
- Parrot;
- Yuneec;
- Others: 3dRobotics, senseFly, Intel…

**Figure 20:**
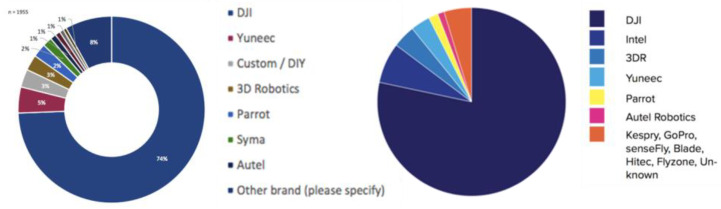
Drone market share.

By 2015, DJI already had 50% of the market share, rapidly rising to 74% [24], by 2017 it had more than 75% of the nonhobbyist drones [25] as shown in Figure 20.

For this reason, DJI was the preferred brand, and among all commercial drones offered by this maker, only the Matrice family offered direct access to the video stream. The 200 series was the only one of all the Matrice family members capable of flying under rain. As custom autonomous navigation and rain support were mandatory, the DJI Matrice 210 was the chosen one.

**Figure 21:**
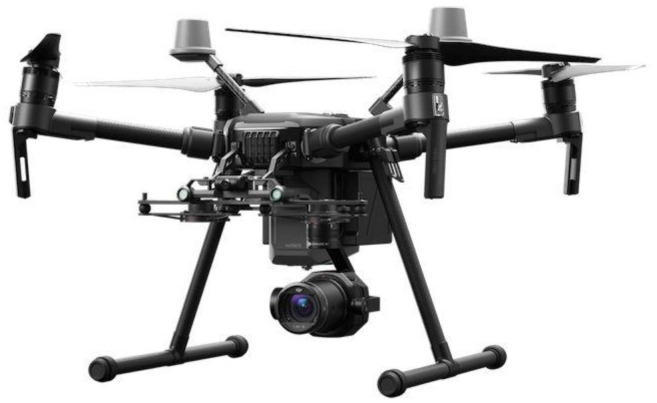
DJI Matrice 210 and Zenmuse 4XS.

The DJI M210 is an 887 × 880 × 378 mm drone complemented with a powerful Zenmuse 4XS camera installed on its 3-axis gimbal (Figure 21).

**Figure 22:**
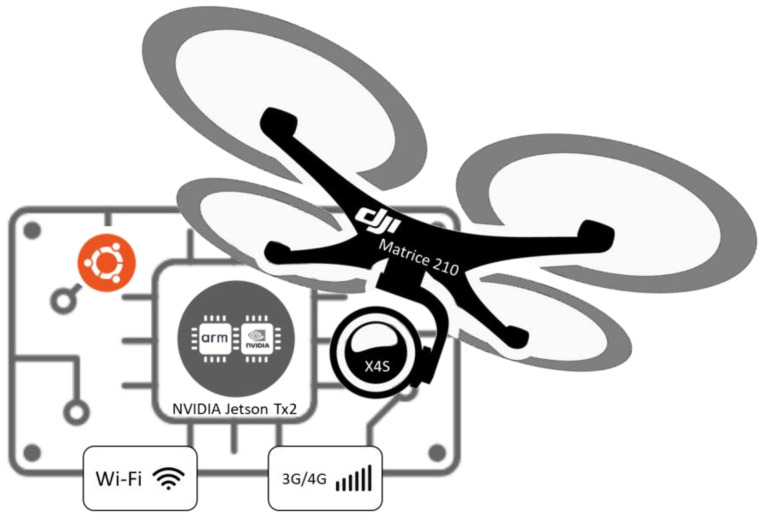
Autonomous drone architecture diagram.

As mentioned before, the autonomous drone system is based on a DJI Matrice 210 and a Zenmuse 4XS camera, but another important component completes the architecture (Figure 22). It is the embedded computer responsible for processing the video stream and control the pilotage.

The embedded computer chosen for the project was an NVIDIA Jetson Tx2—BOXER-8110AI. It provides the required IO connectivity and processing power (thanks to an NVIDIA CUDA) in a compact size. This computer has been equipped with Wi-Fi and 3G/4G interfaces to maintain reliable communication with the ground station.

**Figure 23:**
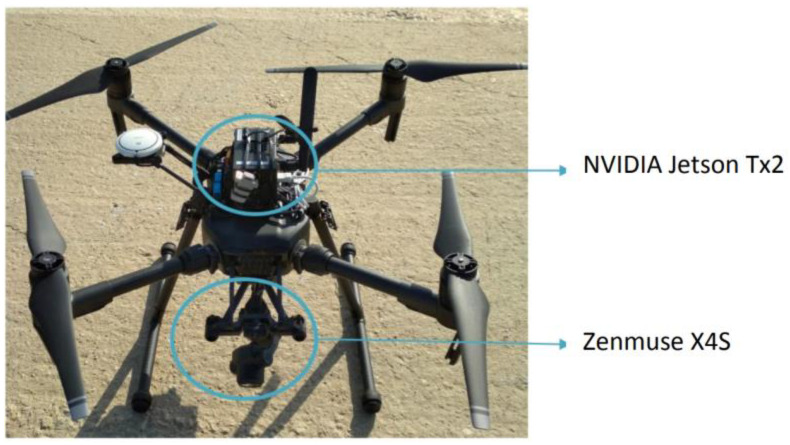
Drone and onboard computer assembly.

The Jetson Tx2 is assembled in the drone and connected to it through only three cables, as shown in Figure 23:

- Power cable is a standard power cord running from the external power connector provided by the drone to the power supply connector of the computer.
- Video cable is a standard USB cable running from the USB provided by the drone to one of the USB host ports of the computer.
- Control cable is a USB to UART TTL cable used to control the drone through DJI’s SDK, and it runs from the TTL port of the drone to any USB host port of the computer.

### 5.2. Algorithm

The embedded computer is running an Ubuntu OS, and its communications are secured with SSH to prevent hackers from manipulating the navigation system or even taking control of the drone.

The system also has the standard remote control supplied by DJI. This allows the change to manual control if required by the security guards at the monitoring stations. There is also a return to home possibility to cancel the flight at any time.

As mentioned, the Jetson TX2 is in charge of running the entire software for the drone system, establishing continuous communication with both the drone autopilot and the ground station. Furthermore, other processes related to intruder detection and tracking, mission control or autonomous movement simultaneously run in the onboard computer.

To provide a clean and effective implementation of the navigation system, the software has been written using the following set of programming languages and libraries:

- CUDA—This library provides access to the parallel execution capabilities of the NVIDIA GPU of the onboard computer.
- ROS Kinetic—This library provides an easy framework and executes concurrent processes in a coordinated and synchronous way.
- OpenCV—Library for image processing (works with CUDA).
- DJI SDK—Library to stable communications with the autopilot of the drone.

**Figure 24:**
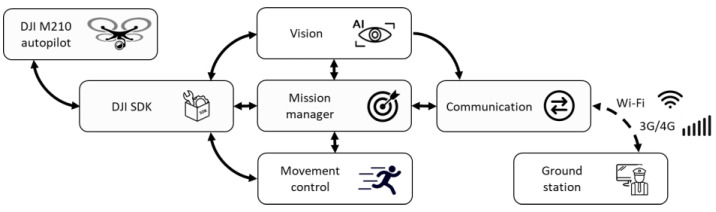
Software blocks.

The software blocks deployed on the onboard computer can be synthesized in Figure 24.

- *DJI SDK*. This node works as a communication bridge, deserializing the autopilot messages (flight data, images, drone status), converting them into a format manageable by the rest of the blocks. It also sends the action commands from the onboard system to the autopilot.
- *Vision*. This block receives all the images and flight data, processes them and detects the intruder. It also reports to the mission manager.
- *Mission manager*. It implements the high-level state machine previously shown in Figure 19, controlling the operation of the mission at all times and taking the appropriate actions in case a pilot is required to take over and manually control the aircraft.
- *Movement control*. This node is in charge of the control, calculating the action commands that must be sent to the autopilot depending on the state of the mission in which the system is at a specific moment.
- *Communications*. It establishes and maintains communications with the ground station. It processes external alarm signals and activates the entire system when required. In parallel, it encodes the images and flight data to send it to the ground to monitor accordingly.

**Figure 25:**
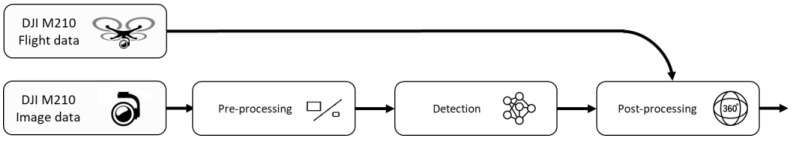
Drone vision algorithm diagram.

The computer vision algorithm runs the pipeline shown in Figure 25.

The input data is the flight and image data, and the output is images plus location information relative to the image and absolute.

The process involved in the algorithm are:

- **Preprocessing**: It receives the images from the autopilot. These images are way too large to be processed by the onboard computer at the frame rate provided. Therefore, the preprocessing performs downscaling to 416 × 416 pixels and provides one image each 50 ms approximately. It also applies the image correction required, with the parameters obtained with the calibration tool, to make sure all image distortion goes away.
- **Detection**: Unlike the video from the static camera network, the drone camera remains in constant motion during the intruder detection and tracking process, so the algorithm based on background subtraction cannot be applied. Given the recent developments in object recognition, which are outperforming classic approaches, such as [26,27], the use of a deep neural network that analyzes each video frame has been chosen. Specifically, the YOLO V3-Tiny [28] architecture was implemented and the retraining of the network from the initial weights provided by the author was done. To work with this network, we used Darknet, which is an environment created by the original authors of YOLO. It is implemented in C language and uses the CUDA library, which uses graphics card resources to operate quickly and efficiently.
  YOLO V3-Tiny is made up of a total of 23 layers, 13 of them being convolutional. As input, it receives an image of size 416 × 416 × 3, which is a color image with three channels. In the output layer, a vector is generated indicating the regions of interest where the people detected are located and the probability associated with each one.
- **Tracking**: In this stage, images are synchronized with the flight data so that each frame can be associated with its flight height and position. Once the intruder has been detected and the image pixels where it is located are known, a tracking stage is performed so that the drone continuously keeps the intruder in the center of the acquired image. A proportional controller is used, which continuously calculates the distance in pixels between the intruder and image center. The output of that controller is converted into speed commands for the drone, which is moved accordingly. A hysteresis cycle was implemented to avoid continuous drone movements when the intruder is very close to the center of the image.

## 6. Experimental Results

### 6.1. CEMF Results

A set of marauding or intrusion events were carried out to create a dictionary of real data. This data was organized and fed to a virtual environment used to validate each version of the algorithm. It was run through real data just acquired by a real sensor as if the event was happening. Thanks to this approach, hundreds of hours of marauding and jumping over a fence were saved. This fact also allowed us to benchmark different implementations over precisely the same circumstances.

**Figure 26:**
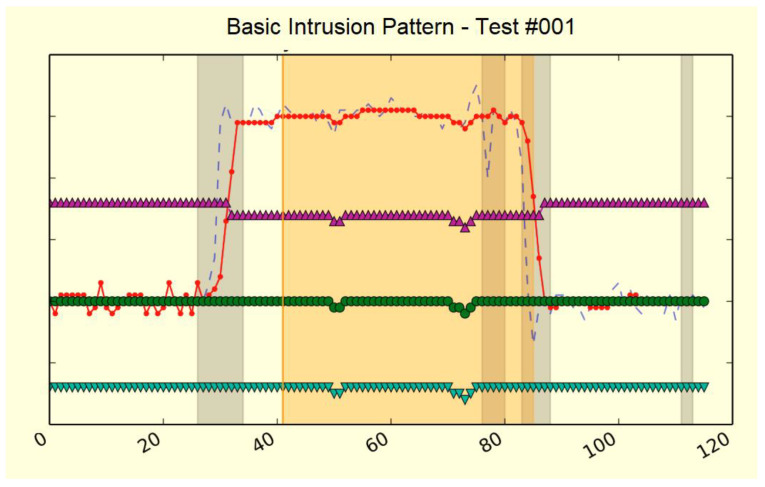
Event sample from the CEMF dataset.

Figure 26 shows the detail of one of the tests included in the test dictionary. Red shows raw CEMF signal, light blue shows filtered signal, green shows the baseline signal, while the other two are internal threshold limits used by the traditional algorithm. To flag where real events occurred, a color code of vertical background bands was used. In the current image, grey bands indicated noisy fragments and an orange band indicated preventive.

**Figure 27:**
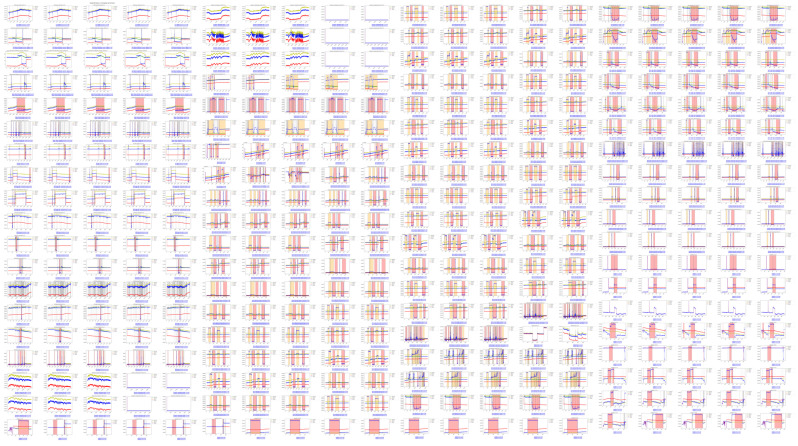
Overview of the CEMF sensor validation’s library.

A JSON file associated with each of the events identifies what type of event should be detected at each sample of the associated stream of CEMF data (and what should not be detected). This is used to launch all tests with a simple command, and so the verification framework can automatically calculate how accurate each implementation is. In Figure 27, the representation of the execution run displaying over 250 individual test plots like the one displayed in Figure 26. This image does not aim to provide details of the result execution but to provide a sense of the magnitude of the validation library.

Once the implementation passed all test cases, the system was deployed in the field. At this point, the proposed algorithm needs to be executed on a low-power microcontroller with reduced computational capabilities. Therefore, the processing task needs to be very optimized, especially if the execution of these tasks is performed in real-time.

In order to validate that the units were operating as expected, a healthcare service was programmed so that the data and the algorithm debug information is permanently recorded in an Influx DB. An automated process was created to help validate the system. It was executed every day to analyze the detected events of the previous day. It generated an image with a data slice that showed how the CEMF signal evolved around each detected event.

**Figure 28:**
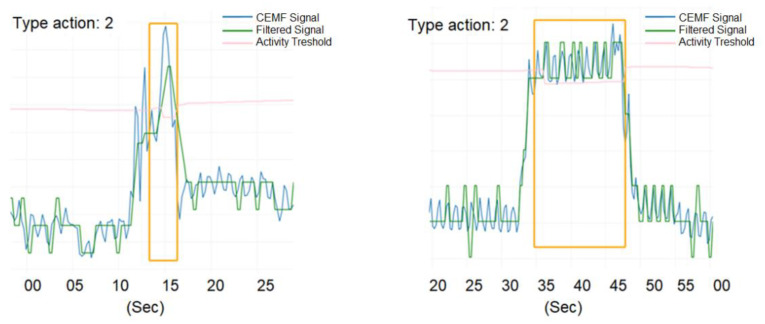
Validation events on the field.

Figure 28 shows two captures corresponding to prowling events. Blue and green are raw and filtered CEMF signals, pink is a preventive threshold of the traditional algorithm, and the yellow rectangle highlights the time while the device detected the threat.

These images are precisely labeled with sensor ID, CEMF channel and timestamped. This fact allows for a manual cross-verification using the video system to validate the cause of the event.

**Figure 29:**
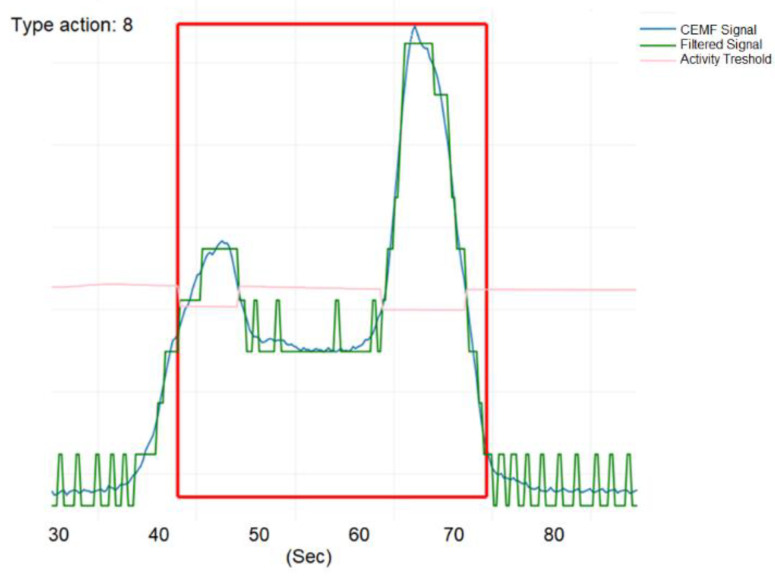

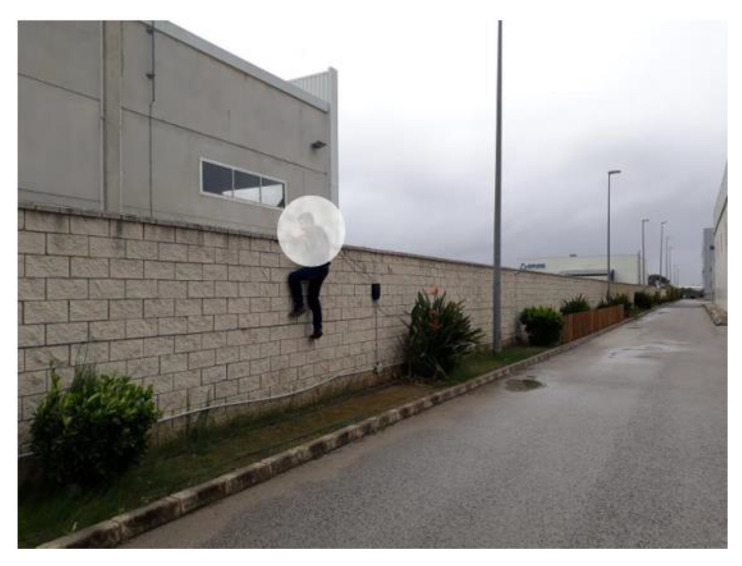
Image validating of a CEMF event in the field.

Figure 29 shows the signal of a person jumping over the fence (green) as well as the filtered (blue) and a traditional threshold (pink). As a result, an intrusion event was detected and highlighted within a red rectangle in the automatically generated images.

**Figure 30:**
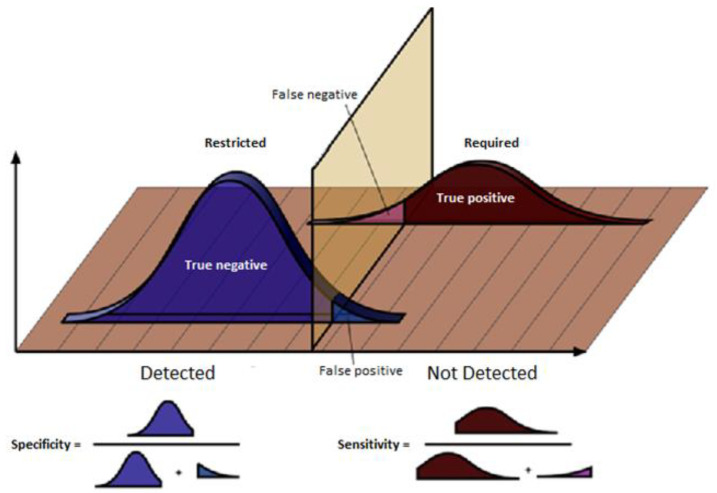
Sensitivity and specificity diagram.

An important part of this field validation was to estimate the performance of the CEMF sensors system. Figure 30 shows the sensitivity and specificity diagram. In order to do so, two different units and three different days were chosen:

- Day 1—This was a windy day that caused the signal of these devices to be altered by external agents such as vegetation in the area. None of these phenomena should be detected by either of the two units. The system should only issue an alarm message if a person is in the perimeter. Therefore, this day will be used to see the robustness of the system against external agents.
- Day 2—During this day a rain shower fell on the site. The CEMF signal is sensitive to rain, so it is crucial to observe the response of each unit under rain conditions. As with the agents mentioned above, rain is also an external agent that should not trigger any alarm.
- Day 3—This was a test day where several persons were asked to perform prowling and intrusion events on each of these two units. This day will be used to validate the success rate of each system against real events.

In summary, the first two days were used to validate the robustness of the system against external agents that may generate false positives, and the last day to assess whether it was able to detect all the events that take place and whether, once detected, it classified them correctly.

As a result of the above analysis, the following statistics and quality indices were obtained:

- True positives (TP): the number of ranges of the ground truth in which a certain event was required and it was effectively detected.
- True negatives (TN): the number of ranges of the ground truth in which a certain event was restricted and it coincided that it was not detected.
- False positives (FP): the number of ranges of the ground truth in which a certain event was restricted and, instead, it was detected.
- False negatives (FN): the number of ranges of the ground truth in which a certain event was required and, instead, it was not detected.
- Sensitivity (Sen): was the proportion of ground truth event ranges that were correctly classified as detected from among all those that were required.
- Specificity (Spec): is the proportion of ground truth event ranges that were correctly classified as undetected from among all those that were restricted.
- Accuracy (Acc): is the proportion of total hits, without distinguishing between true positives and true negatives.

The respective formulas that allow the calculation of the quality indices described above are:

(9) $$Sen=\frac{TP}{TP+FN}$$

(10) $$Spec=\frac{TN}{TN+FP}$$

(11) $$Acc=\frac{TP+TN}{TP+TN+FP+FN}$$

As can be seen in Table 2, the closer these quality indices are to 100%, the more reliable the algorithm is, since it means that it allows better discrimination between the two populations of required and restricted events. In particular, the overall performance of the system across the validation days was very good. With a sensitivity, specificity and accuracy over the 95% it proved that the system is very robust against false alarms and very accurate for true alarms.

### 6.2. Vision Results

In order to work with neural networks, an initial training step is required for the network to learn the features of the objects that need to be detected. To perform the training most optimally, a large number of images that correctly represent the target problem that the network must solve are required for this process. In this project, we counted on a large number of aerial images in which people were present. In addition, to carry out the training with these images, they had to have the associated metadata to flag the pixels where persons appeared in each frame.

**Figure 31:**
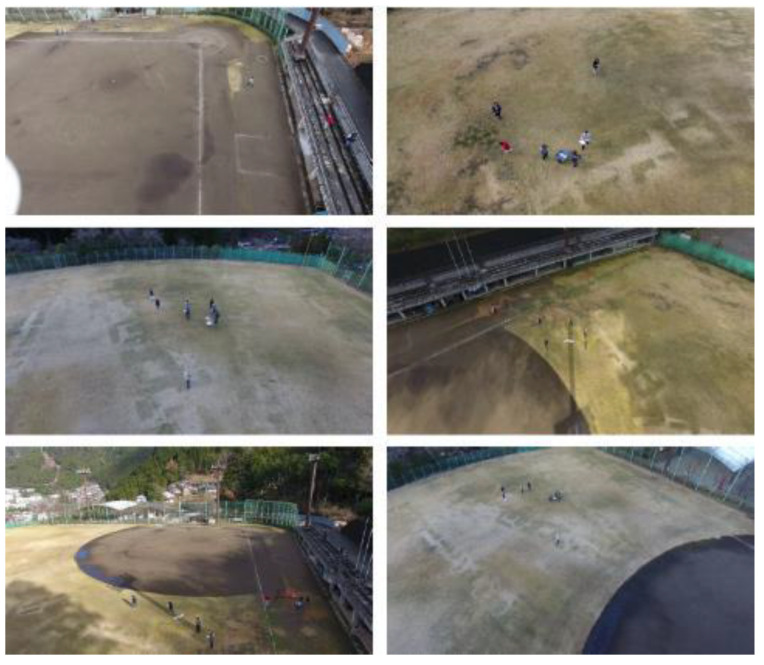
Dataset sample images.

For training the network, a data set was created using a combination of downloaded public datasets and multiple images of people acquired by our drone at different heights, with a camera tilt of approximately 45° pointing towards the ground. The ratio of public images to own images was approximately 85/15. In the case of the public dataset, the Okutama Action dataset [29] was used. Approximately 60,000 images were used, separating 48,000 images for training and 12,000 images for validation (checking that the network worked as expected). In this way, the 80% training—20% validation criterion was followed. Figure 31 shows some samples of this dataset.

**Figure 32:**
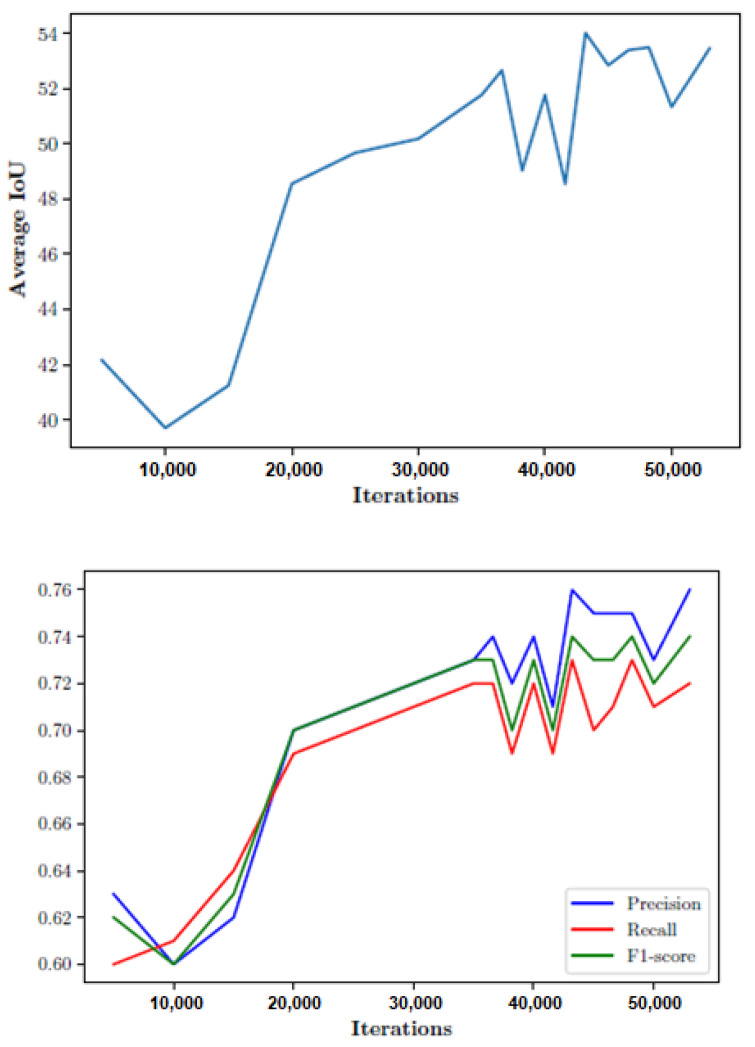
(**Top**) Average IoU and precision (**bottom**).

During the network training, several metrics were analyzed in each iteration, such as intersection over union (IoU) and the precision, recall, F1-score. By analyzing the validation set in each complete iteration of the network training, the graphs in Figure 32 were obtained. As can be seen, the algorithm behaved quite well for the validation set, evolving positively to stay above 50% in the case of the IoU. On the other hand, the precision, the recall, and the F1-score equally evolved positively until settling in the range of 0.70–0.76. It should be noted that, when detecting people for the scope of this project, the point of interest may not be to achieve a great precision when obtaining the area of interest marking a person, but rather that the number of false positives and false negatives are kept as low as possible.

**Figure 33:**
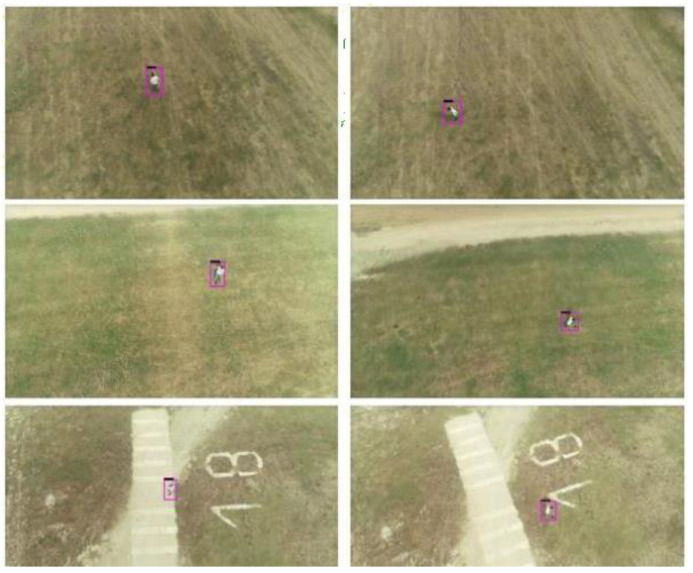
Inference results sequence for one person.

**Figure 34:**
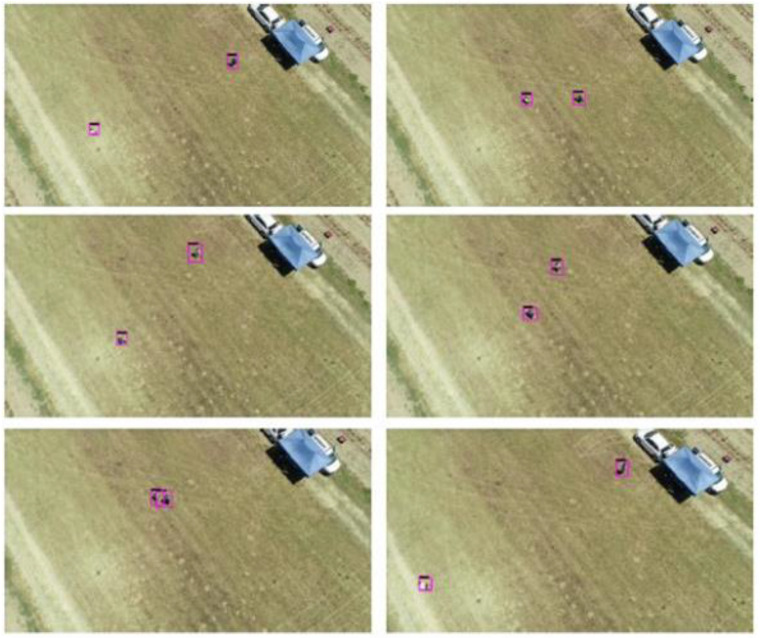
Inference results sequence for two people.

Several flights were made at different heights between 15 and 30 m above the ground to validate the implementation. People appeared and moved in realistic situations, as can be seen in Figure 33 and Figure 34. It can be seen how the detected people are marked with a purple region of interest.

## 7. Conclusions

A complete perimeter electronic security system has been proposed to identify dangerous intrusions based on disruptive CEMF sensor technology and video information. The surveillance system exhibits long-distance monitoring, high sensitivity, and reduced false alarms since the CEMF technology has a high detection range and can discriminate between animals, humans, and objects. The technology is developed to accurately detect the motion of people in a protected area in an outdoor environment. It provides intelligent mechanisms for reducing false alarms and improving the security system’s effectiveness in detecting and preventing potential intrusions. The system also includes cameras and video content analytics for automatic intruder tracking. Additionally, the advantageous properties of the CEMF sensors provide reduced false alarms and allow for the cameras to be deactivated most of the time. A mobile camera on an autonomous drone system allows better intruder detection and tracking when the threat is out of range of the perimeter units and the static ground cameras.

## Figures and Tables

**Table 1:** Ensemble performance table.

	Idle Class 1	Prowling Class 2	Intrusion Class 3	Car Class 4	% Accuracy
**Idle**	93	1	0	0	98.9
**Prowling**	9	114	0	0	92.7
**Intrusion**	0	0	108	0	100
**Car**	0	0	0	106	100

**Table 2:** Sensitivity and specificity of the CEMF system.

		Day 1	Day 2	Day 3
−	True negative	19	26	20
Prowling	True positive	5	2	29
	False negative	0	0	0
	False positive	1	2	0
Intrusion	True positive	0	0	14
	False negative	0	0	0
	False positive	0	0	0
Car	True positive	3	4	5
	False negative	0	1	0
	False positive	0	0	0
Total	True negative	65		
	True positive	62		
	False negative	1		
	False Positive	3		
Sensitivity		98%		
Specificity		96%		
Accuracy		97%		

## Data Availability

Not applicable.
